# Coronavirus Disease 2019 Vaccine-Induced Flare-Up of Severe Bronchial Asthma Previously Controlled With Dupilumab: A Case Report

**DOI:** 10.7759/cureus.38122

**Published:** 2023-04-25

**Authors:** Toshiyuki Sumi, Kentaro Kodama, Hirotaka Nishikiori, Yusuke Tanaka, Hirofumi Chiba

**Affiliations:** 1 Respiratory Medicine, Hakodate Goryoukaku Hospital, Hakodate, JPN; 2 Respiratory Medicine and Allergology, Sapporo Medical University, Sapporo, JPN; 3 Respiratory Medicine and Allergology, Sapporo Medical University School of Medicine, Sapporo, JPN

**Keywords:** vaccination, covid-19, dupilumab, severe asthma, flare-up

## Abstract

The widespread after-effects of the coronavirus disease 2019 (COVID-19) are still a grave threat worldwide. Among them are adverse reactions to vaccines, including those most observed following Pfizer-BioNTech (BNT162b2) vaccine administration, namely, local reactions at the injection site, fatigue, headache, myalgia, chills, arthralgia, and fever. Patients with asthma particularly present with unique adverse reactions to the BNT162b2 vaccine, notably, an exacerbation in their asthma symptoms as highlighted through the current case report. In this case, a 50-year-old woman had been undergoing treatment for bronchial asthma in the form of inhalation steroids and dupilumab, as well as systemic steroid prednisolone as maintenance therapy. She had mild injection site reactions after her first three COVID-19 vaccinations. She also experienced acute exacerbation requiring hospitalization after the fourth and fifth doses. Her symptoms resolved following steroid therapy. The close association between the timing of vaccinations and the onset of clinical symptoms suggests that the exacerbation episodes were triggered by the vaccine. Therefore, although the BNT162b2 vaccine is safe to administer in patients with bronchial asthma, cases reporting patients sensitized to the BNT162b2 vaccine developing bronchial asthma or experiencing asthma exacerbations should not be neglected. Clinicians should be aware of the possibility of flare-ups induced by repeated COVID-19 vaccinations in such patients.

## Introduction

The most common adverse reactions to the BNT162b2 vaccine (Pfizer-BioNTech, New York, NY) are local reactions at the injection site, fatigue, headache, myalgia, chills, arthralgia, myocarditis, thrombosis, and fever. Serious allergic reactions include anaphylaxis [1]; however, the incidence is very low, at 4.8 per million doses [2], and other allergic reactions are unknown. The BNT162b2 vaccine is safe to administer in patients with bronchial asthma; however, case reports have been published regarding patients sensitized to the BNT162b2 vaccine developing bronchial asthma or experiencing asthma exacerbations [3,4]. The patient in this present study had severe bronchial asthma that was controlled with a biologic agent and developed recurrent asthma exacerbations after the fourth and fifth doses of the BNT16b2 vaccine. Asthma exacerbation may be a unique adverse reaction in patients with asthma due to sensitization to the BNT162B2 vaccine. Herein, we report this experience to help determine its occurrence and relevant treatment.

## Case presentation

The patient was a 50-year-old woman weighing 82 kg with severe bronchial asthma, treated and controlled with fluticasone furoate/vilanterol trifenatate/umeclidinium bromide (100 µg/62.5 µg/25 µg) and dupilumab (300 mg) every two weeks, and systemic steroid prednisolone (5 mg/day). She had a history of atopic dermatitis and was diagnosed with bronchial asthma at the age of 24 years, which was treated with dupilumab from the age of 46 years. After starting dupilumab her symptoms stabilized, and she had no flare-ups. She received three coronavirus disease 2019 (COVID-19) vaccinations (BNT162b2, BNT162b2, and mRNA-1273 (Moderna, Cambridge, MA)), all with only mild injection site reactions. There was no change in the systemic steroid dosage before or after the patient’s vaccinations, and there was no pre-vaccination drug withdrawal. In September 2022, the patient visited the emergency room because of a persistent cough and wheezing beginning 12 hours after the fourth dose of the BNT162b2 vaccine. Laboratory tests revealed an elevated white blood cell count (12,000/μL), loss of eosinophil count (0/μL; 0%), and no elevation in the C-reactive protein level (0.10 mg/dL). Blood gas analysis showed hypoxemia, with a pH of 7.422, pO2 of 56 mmHg, and pCO2 of 38.2 mmHg (on room air). On physical examination, wheezing was detected in both the lungs, without any heart murmurs. Repeated inhalation of short-acting β2-agonists and intravenous methylprednisolone did not improve her symptoms. The patient was diagnosed with a bronchial asthma flare-up and was hospitalized for 10 days with intravenous steroid administration (methylprednisolone 120 mg/day). Her symptoms subsequently stabilized, and she was discharged.

**Figure 1:**
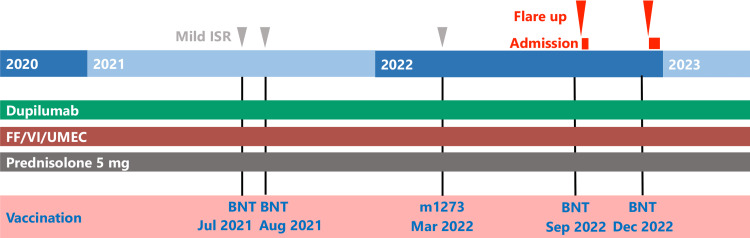
Clinical course of COVID-19 vaccine-related adverse reaction events The patient had mild ISRs after the first three vaccinations. Following the fourth and fifth COVID-19 vaccinations, the patient had asthma flare-ups, both of which resulted in hospitalization. COVID-19, coronavirus disease 2019; ISR, injection site reaction; FF/VI/UMEC, fluticasone furoate/vilanterol trifenatate/umeclidinium bromide; BNT, BNT162b2

However, in December 2022, she received her fifth dose of the bivalent BNT162b2 vaccine (original + omicron BA.4/BA.5). Ten hours after the fifth dose, the patient developed bouts of coughing and wheezing and had to be urgently admitted to the hospital. Laboratory tests revealed an elevated white blood cell count (11,800/μL), a decreased number of eosinophils (35.4/μL; 0.3%), and a mildly elevated C-reactive protein level (0.35 mg/dL). She was treated with intravenous steroids (methylprednisolone 120 mg/day) for 14 days and was discharged following resolution of symptoms. In both exacerbation episodes, none of the imaging findings suggested bacterial or viral infection, and the patient’s sputum and blood cultures were negative. The severe acute respiratory syndrome coronavirus 2 (SARS-CoV-2) polymerase chain reaction test result was also negative. The patient’s diagnosis was bronchial asthma flare-up induced by vaccine administration; the course of events is presented in Figure 1. Her asthma symptoms stabilized post-discharge, and her systemic steroid dosage was restored to the maintenance dose (prednisolone 5 mg/day). Informed consent was obtained from the patient for reporting this case.

## Discussion

In the current case, our patient, who had severe bronchial asthma and was on biologic therapy, experienced similar courses of bronchial asthma exacerbation after the fourth and fifth doses of a COVID-19 vaccine. The vaccine probably triggered the bronchial asthma exacerbation, based on the close association between the vaccination timelines and the onset of the clinical symptoms.

The BNT162b2 vaccine induces high levels of a specific cluster of differentiated CD4+ T cells that primarily produce T helper 1 (Th1) cytokines, as opposed to T helper 2 (Th2) cytokines [5]. Th2-type disorders caused by the BNT162b2 vaccine appear to be rare, although several case reports have been published regarding Th2-type disorders associated with eosinophils, including eosinophilic polyangiitis granulomatosis [6]. Three previous studies have reported COVID-19 vaccine-induced asthma exacerbations (Table 1) [3,4,7]. In two of these reports, similarly to ours, bronchial asthma worsened the day after vaccination, which suggests a delayed allergic reaction (lymphocytic involvement) [4,7]. Ando et al. also suggested sensitization to the vaccine based on an increase in peripheral blood eosinophils after vaccine administration [4]. However, our patient had no change in eosinophil levels after either of the vaccine administrations. One of the possible explanations could be that the increase in eosinophils was masked because the patient was on continuous oral prednisolone therapy. Another possible cause of asthma exacerbation may be allergic reactions to the excipient polyethylene glycol (PEG). [8] The two prior BNT162b2 vaccine doses might have sensitized the patient to PEG, and the presence of anti-PEG antibodies might have caused the asthma exacerbations following the fourth and fifth COVID-19 vaccinations.

**Table 1:** COVID-19 vaccine-induced asthma exacerbations ND; not described

Case	Sex	Age	Past history	Phenotypes of asthma	Smoking history	Time from vaccine administration to asthma exacerbation	Vaccine name	Number of vaccine doses	WBC	Eosin％	Reference
1	female	28	Bronchial asthma, allergic rhinitis	ND	Former	3 weeks	BNT16b2	2	ND	ND	[3]
2	female	55	Marfan syndrome, sinusitis	Eosinophilic	ND	One day	BNT16b2	3	10240	19.4	[4]
3	female	76	Bronchial asthma, hypertension, diabetes	ND	ND	One day	CoronaVac	1	12600	9	[7]
4	female	50	Bronchial asthma, atopic dermatitis	atopic	Never	One day	BNT16b2	4 and 5	11800/12000	0/0.3	Our patient

Data on severe asthma associated with COVID-19 are limited. The incidence of COVID-19 in patients with severe asthma is relatively low and not associated with a high risk of SARS-CoV-2 infection or poor outcome [9]. Based on data from patients in the Severe Asthma Network in Italy, scientists concluded that severe asthma was not an independent risk factor for severe COVID-19 [10], although chronic systemic steroid users (oral prednisone, 5-40 mg/day) have been reported to have increased COVID-19 severity and increased mortality [11]. Our patient was a chronic steroid user; therefore, the COVID-19 vaccine was recommended in this situation. No increased risk of SARS-CoV-2 infection exists during treatment with dupilumab, and the administration of the COVID-19 vaccine is safe and well-tolerated [12]. The biologic drug used in our patient, dupilumab, is a humanized monoclonal antibody that targets the alpha chain of the interleukin (IL)-4 receptor, which is common to IL-4 and IL-13. In a report on the safety and tolerability profile of the mRNA SARS-CoV-2/COVID-19 vaccine in patients with severe asthma receiving biologic therapy, the asthma control test scores increased with vaccination, regardless of the type of biologic agent used, and no exacerbation of asthma occurred [12]. Based on this information, it is unlikely that dupilumab had an unfavorable effect on the asthma exacerbation caused by the BNT162b2 vaccine in our case.

## Conclusions

COVID-19 vaccination was recommended for our patient because she had severe bronchial asthma and was a chronic oral prednisolone user. However, clinicians should be wary of the possibility of flare-ups induced by COVID-19 vaccines in such patients. It is, therefore, necessary to accumulate cases and investigate the mechanism of asthma exacerbation caused by COVID-19 vaccination.
